# A Method for Few-Shot Radar Target Recognition Based on Multimodal Feature Fusion

**DOI:** 10.3390/s25134162

**Published:** 2025-07-04

**Authors:** Yongjing Zhou, Yonggang Li, Weigang Zhu

**Affiliations:** Department of Electrical and Optical Engineering, Space Engineering University, Beijing 101416, China; yongjing_zhou@hgd.edu.cn (Y.Z.); liyonggang@hgd.edu.cn (Y.L.)

**Keywords:** few-shot learning, multimodal fusion, target recognition, natural resonant frequency

## Abstract

Enhancing generalization capabilities and robustness in scenarios with limited sample sizes, while simultaneously decreasing reliance on extensive and high-quality datasets, represents a significant area of inquiry within the domain of radar target recognition. This study introduces a few-shot learning framework that leverages multimodal feature fusion. We develop a cross-modal representation optimization mechanism tailored for the target recognition task by incorporating natural resonance frequency features that elucidate the target’s scattering characteristics. Furthermore, we establish a multimodal fusion classification network that integrates bi-directional long short-term memory and residual neural network architectures, facilitating deep bimodal fusion through an encoding-decoding framework augmented by an energy embedding strategy. To optimize the model, we propose a cross-modal equilibrium loss function that amalgamates similarity metrics from diverse features with cross-entropy loss, thereby guiding the optimization process towards enhancing metric spatial discrimination and balancing classification performance. Empirical results derived from simulated datasets indicate that the proposed methodology achieves a recognition accuracy of 95.36% in the 5-way 1-shot task, surpassing traditional unimodal image and concatenation fusion feature approaches by 2.26% and 8.73%, respectively. Additionally, the inter-class feature separation is improved by 18.37%, thereby substantiating the efficacy of the proposed method.

## 1. Introduction

In recent years, deep learning and big data technologies, serving as pivotal components within the artificial intelligence domain, have propelled significant advancements in target recognition [1,2] through innovative architectural designs and algorithmic breakthroughs, thereby unlocking the potential for knowledge extraction from vast datasets. However, the strong dependence of the existing supervised learning paradigm on the scale and quality of labeled data severely restricts its application efficacy in scenarios with limited labeling resources. The main applications of radar target recognition technology include disaster monitoring, geological exploration, and camouflage target recognition. The primary recognition object is a non-cooperative target. However, several issues arise, including long data acquisition and processing cycles, unstable and imprecise feature extraction, and high annotation costs and the need for expert knowledge. These issues severely limit the application of big data in traditional deep learning methods. In this context, how to exploit the advantages of few-shot learning (FSL) [3] has become an urgent need for radar target recognition applications. FSL has become a research frontier technology in radar target recognition due to its unique knowledge migration mechanism. By constructing a relocatable feature space and efficient meta-learning strategies, discriminative features with strong generalization ability can be extracted from a very small number of labeled samples, and fast adaptation to new classes of targets can be achieved.

**Figure 1:**
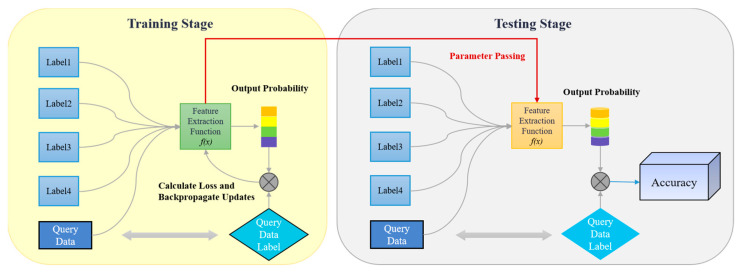
Flowchart of a typical classification task under FSL [13]. After feature extraction, the probability of the sample belonging to each label is calculated by the feature vector and the classifier. In the training stage, parameters is optimized by the loss function. This flowchart is typically used in metric learning based few-shot classification tasks (e.g., prototypical networks or matching networks).

At present, the majority of research in the domain of FSL for radar target recognition is oriented towards the utilization of features such as synthetic aperture radar (SAR) [4,5,6,7,8] and high-resolution range profile (HRRP) [9,10,11]. The investigation of models and features for resonance zone signals remains in the exploratory phase. While optical region features are advantageous in target characterization, the complex nature of feature extraction results in low computational efficiency and unstable features, which hinders its applicability in FSL scenarios. In light of the model’s necessity to extract features exhibiting enhanced differentiation and stability from limited samples, this paper proposes to utilize the natural resonance frequency feature of the resonance region as the core recognition feature. This feature is seen to reflect the intrinsic electromagnetic scattering characteristics of the target in the resonance region, thereby enabling the characterization of the target’s resonance scattering characteristics in a very low dimension. Furthermore, the intrinsic physical properties and scattering mechanism of this feature ensure a certain degree of invariance of the attitude and observation angle [12], which is more compatible with the generalization demand for features in FSL. This theoretical framework provides a robust foundation for the study and its findings. A flowchart of a typical classification task in an FSL scenario according to the literature [13] is presented in Figure 1.

In recent years, the field of computer vision has witnessed considerable advancements in multimodal FSL [14,15,16,17]. However, the majority of extant studies are confined to the integration of features such as optical images, text, and other modalities, lacking a comprehensive fusion mechanism that encompasses the deep physical properties of the target. In the domain of radar target recognition, prevailing methodologies predominantly depend on single-modal features, which are inadequate in addressing the substantial variations in features within complex electromagnetic environments. Furthermore, it is challenging to establish rational cross-modal correlations. In order to further enhance the comprehensiveness of features in scenarios where sample sizes are limited, this study challenges the conventional single-modal feature learning paradigm. It proposes an innovative approach that integrates natural resonance frequencies with signal time-frequency characteristics in a multimodal manner. The proposed multi-mode fusion classification network based on residual neural network and bi-directional long short-term memory (Res-BiLSTM-MFCNet) to achieve cross-modal fusion of time-frequency image features and natural resonance frequency features in radar signals. The energy-guided attentional fusion framework is embedded, and the cross-modal equilibrium loss (CME loss) function is designed based on the composite of feature similarity metrics and classification loss. The objective is to facilitate radar target recognition under small sample conditions.

The primary research contributions and innovations of this paper can be outlined as follows:

1. In response to the shortcomings of traditional meta-learning techniques that emphasize model generalization at the expense of feature representation, a novel methodology is introduced. This approach incorporates the natural resonance frequency sequence, which delineates the scattering characteristics of the target resonance area, into the FSL framework. By capitalizing on the angular and attitude stability inherent in the natural resonance frequency, this method enables the fusion of multimodal features, thereby reducing the influence of extraction accuracy on the natural resonance frequency.
2. The Res-BiLSTM-MFCNet model is introduced, wherein the energy associated with each natural resonant frequency mode is incorporated into the features as a matrix of weight coefficients. This model facilitates the deep integration of multimodal features through a feature mapping process that encompasses encoding, fusion, and decoding, ultimately leading to the desired classification outcome.
3. The CME loss, which integrates a feature similarity measure with classification loss, is intended to enhance the discriminability of the feature space while enabling the model to acquire a more distinct feature representation.

The experimental findings demonstrate that, without a significant increase in inference time and computational complexity, the proposed method achieves a recognition accuracy of 95.36% under varying observation angles. This accuracy is 2.26% and 8.73% higher than that of traditional unimodal and concatenation fusion feature methods, respectively. Additionally, the ablation experiment further validates the effectiveness of each innovative module and confirms the feasibility and practical value of the proposed method.

## 2. Res-BiLSTM-MFCNet

**Figure 2:**
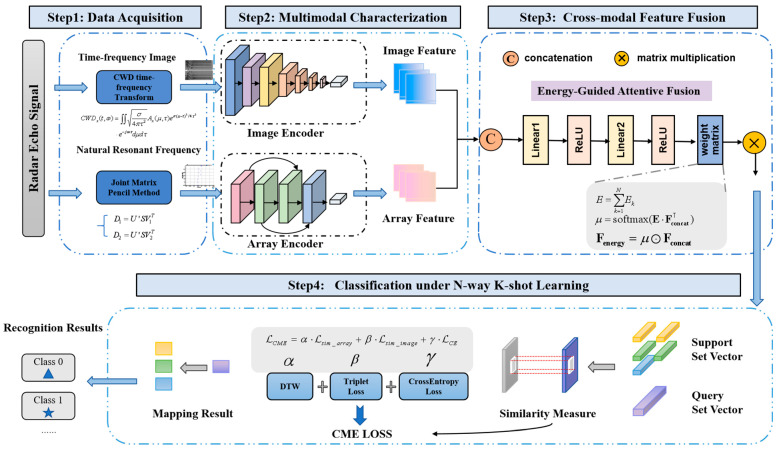
Flowchart of Res-BiLSTM-MFCNet. It mainly consists of four steps: data acquisition, multi-modal representation module, cross-modal fusion module, and small sample classifier. The natural resonance frequency and time-frequency images are extracted from the radar echo signal. Target recognition is achieved by mapping these images through the classifier via an encoder-fusion-decoding process, which is integrated with the EGAF framework.

As illustrated in Figure 2, the Res-BiLSTM-MFCNet model is utilized for recognizing few-shot target tasks $T=\left\{D_{train},D_{test}\right\}$, leveraging both training and test samples $x^{s}\in D_{train}^{i}$, $x^{q}\in D_{test}^{i}$ from a known category dataset $D_{base}$. The model is primarily composed of three core phases: multimodal feature characterization, cross-modal feature fusion and FSL classification.

In the multimodal feature characterization stage, cross-modal cooperative coding processing is developed based on time-frequency image features and natural resonant frequency sequence features. The essence of this process lies in the realization of multidimensional decoupling and reconstruction of the target’s scattering characteristics through the optimization of various feature spaces. The feature extraction framework equips the model with the ability to represent multimodal features effectively. For the time-frequency image features, ResNet-18 is employed to construct the spatial energy distribution extraction module, where energy aggregation features in the time-frequency domain are extracted layer by layer using a multilayered convolutional kernel approach. For the natural resonance frequency sequence features, the oscillatory variation patterns of resonance poles over time and the angular stabilization characteristics are captured using a BiLSTM network. The scattering characteristics of the target are described jointly from the different dimensions of signal energy distribution and resonance oscillation changes, resulting in a joint feature representation with enhanced discriminative properties.

In the cross-modal feature fusion stage, the energy-guided attentive fusion (EGAF) framework is proposed to provide a more comprehensive description of the physical properties of the signal. The model’s ability to characterize the physical nature of the signal is enhanced by the introduction of an energy weight matrix during the projection of the fused features. This matrix intuitively reflects the relative importance of various natural resonant frequency modes within the signal, thereby facilitating the model’s ability to capture and learn the signal’s key features. To address the local feature sensitivity of non-visual signals, the model utilizes a multi-level feature fusion capability to enhance feature sensitivity. This is accomplished by incorporating guidance from the scattering mechanism, which strengthens the feature response in critical frequency bands through the allocation of energy weights. Consequently, this approach improves feature discriminability.

In the FSL classification stage, training sample pairs are constructed from the support set and query set. A weighted combination of triplet loss, dynamic time warping (DTW), and cross-entropy loss is utilized as the CME loss. The triplet loss and DTW serve as similarity measures for image features and sequence features, respectively, enhancing the discriminative properties of the features. Meanwhile, the cross-entropy loss optimizes the model’s classification performance. The DTW loss improves the stability of the natural resonance frequency through elastic temporal alignment, providing a modal complement to the spatial discriminative constraints imposed by the triplet loss. The weighting coefficients are designed to prevent a loss function and to concentrate the optimization on the classification task. Due to the suboptimal extraction quality of the natural resonant frequency features, the image features are assigned a weight that is twice that of the natural resonant frequency features. The components of the loss function correspond to time-domain matching, spatial separability, and classification inference in signal processing, respectively. This design ensures both the model’s ability to distinguish between inter-class samples and its classification accuracy. A 5-way 1-shot few-shot classification sampling strategy, which is a more common approach, is employed to estimate the correlation of inter-sample features for classification purposes, thereby facilitating the completion of the target recognition task.

### 2.1. Multimodal Characterization Module

The multimodal feature characterization module primarily comprises a time-frequency image encoder and a natural resonance frequency sequence feature encoder. The time-frequency image encoder constructs a time-frequency capability distribution parsing module based on ResNet-18 [18]. Its set of convolution kernels extracts the time-frequency features $F_{\mathrm{img}}$ of the signal layer by layer. We establish the characteristic expression formula as follows:

(1) $$F_{\mathrm{img}}=\mathrm{ResNet}(x_{CWD};\theta_{\mathrm{ResNet}})$$

$x_{CWD}$ is the time-frequency image generated by the CWD time-frequency transform and θResNet is the network parameter.

BiLSTM [19] temporal encoder is designed for natural resonance frequencies to capture the contextual dependencies of feature sequences $F_{\mathrm{seq}}$ through forward and backward hidden states $\vec{h_{t}}$ and $\overset{\leftarrow }{h_{t}}$, specifically for natural resonance frequencies. We establish the characteristic expression formula as follows:

(2) $$F_{\mathrm{seq}}=\mathrm{BiLSTM}(x_{\mathrm{Res}};\theta_{\mathrm{BiLSTM}})$$

$x_{\mathrm{Res}}\in R^{1\times d}$ is the natural resonance frequency sequence and d is the length of the sequence.

**Figure 3:**
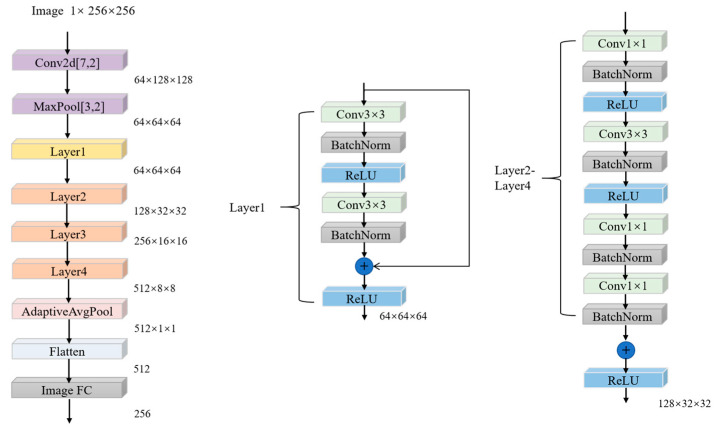
The structure diagram of the image encoder illustrates that layer 1 has the simplest configuration, consisting solely of two 3 × 3 convolution modules, a regularization component, and a ReLU activation function. Layers 2 through 4 share the same structure, which can be viewed as an extension of the first layer.

Input single-channel time-frequency grayscale image $X_{\mathrm{img}}\in R^{1\times 256\times 256}$, and its processing flow is as follows:

The initial convolutional layer utilizes a large-scale receptive field convolutional kernel to extract features related to low-frequency energy distribution. The configuration of this kernel is as follows: kernel size = 7 × 7, stride = 2, padding = 3. Consequently, an output feature map is generated. Following this, a normalization and activation process is initiated, maintaining the full dimensionality. The residual network layer group is responsible for feature abstraction through a four-level residual structure. Layer 1 consists of two standard residual blocks, where the convolution operation is denoted by Fout=ReLU(BN(Conv(Fin)))+Fskip. $Conv(F_{in})$ denotes the convolution operation on the input features $F_{in}$, BN stands for batch normalization, ReLU is rectified linear unit function and $F_{skip}$ is skip connection. $F_{out}$ stands for output feature and the output dimension is 64 × 64 × 64. The remaining three layers consist of two bottleneck residual blocks with downsampling. Each block follows a sequence of 1 × 1 downsampling, a 3 × 3 convolution, and another 1 × 1 downsampling. The residual blocks are adjusted to match the dimensions using a stride of 2, resulting in output dimensions of 128 × 32 × 32, 256 × 16 × 16, and 512 × 8 × 8. Finally, global mean pooling and fully connected layers are employed for feature compression and dimensionality reduction, yielding image features with 256 dimensions. The structure of the image encoder is illustrated in Figure 3, which is followed by a pooling layer with a kernel size of 3 × 3, a stride of 2, and padding of 1, resulting in the final output.

Input the natural resonant frequency imaginary part sequence $X_{\mathrm{seq}}\in R$, the processing flow is as follows:

The time-series embedding layer performs a linear projection with an output dimension of 64, while each hidden cell of the two-layer BiLSTM has 128 dimensions. The embedding layer maps the scalar sequence to a higher-dimensional space, thereby enhancing its nonlinear characterization. The BiLSTM network has the capacity to represent both the forward resonance build-up and the backward resonance decay processes through forward and backward propagation, respectively. According to [19], the forward propagation formula is:

(3) $$\vec{h_{t}}=LSTM(\vec{h_{t-1}},H_{0}^{\left(t\right)};\theta_{forward})$$

$\vec{h_{t}}$ is the hidden state at the current time step t and denotes the output of the forward LSTM at time step t. $\vec{h_{t-1}}$ is the hidden state of the previous time step t−1 and serves as one of the inputs for the current time step. The variable $H_{0}^{\left(t\right)}$ is the input feature at time step t, while $\theta_{forward}$ is the set of parameters for the forward LSTM.

The backward propagation formula [19] is:

(4) $$\overset{\leftarrow }{h_{t}}=LSTM(\overset{\leftarrow }{h_{t+1}},H_{0}^{\left(t\right)};\theta_{backward})$$

$\overset{\leftarrow }{h_{t}}$ represents the output of the backward LSTM at time step t, $\overset{\leftarrow }{h_{t+1}}$ is the hidden state of the later time step t+1 and serves as one of the inputs to the current time step. The variable $\theta_{backward}$ is the set of parameters of the backward LSTM, which has a similar structure to the forward LSTM, but the parameter values may be different.

**Figure 4:**
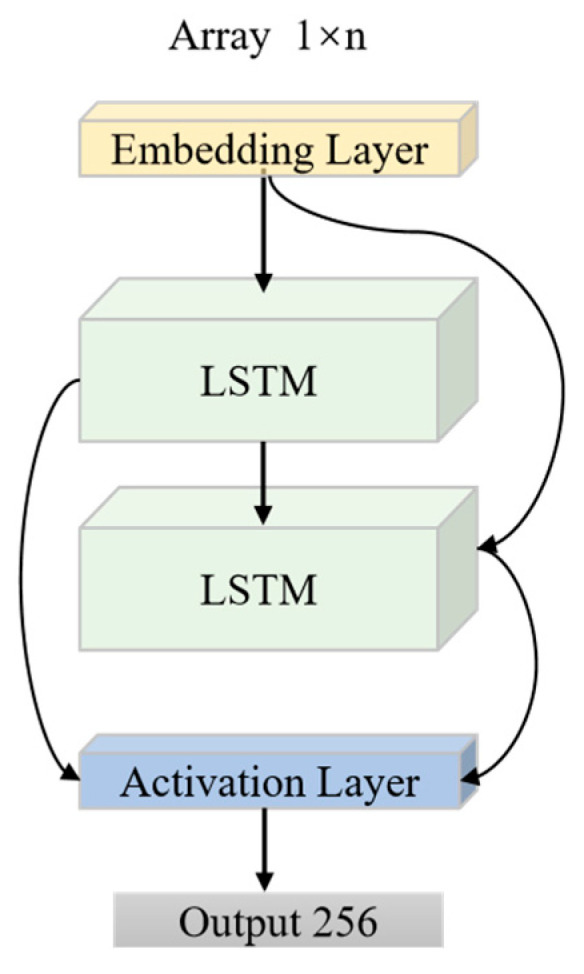
The structure of the natural resonant frequency encoder begins with an embedding layer that transforms the input sequence data into a dense vector. A bidirectional long short-term memory (LSTM) layer then processes the sequences sequentially over time to capture temporal dependencies. Following this, the activation layer applies a nonlinear transformation to the output of the LSTM. The final output is a 256-dimensional feature vector, which serves as the global representation of the sequence.

Finally, the features are spliced to output a 256-dimensional feature vector. The image encoder structure is shown in Figure 4.

In this paper, two networks are selected for feature extraction and fusion. To address the local feature sensitivity of non-visual signals, the model’s multi-level feature fusion capability is utilized. Additionally, the guidance of the scattering mechanism is incorporated to enhance the feature response of key bands through dynamic weight allocation, thereby improving the discriminability of the features.

### 2.2. Cross-Modal Feature Fusion Module

**Figure 5:**
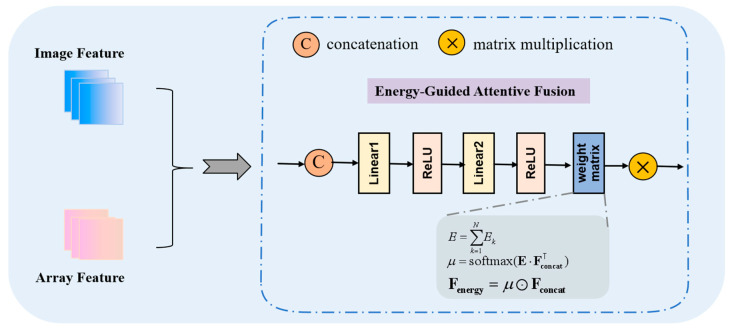
Flowchart of cross-modal feature fusion. The image features and array features are concatenated along the specified dimension to create a multimodal joint feature representation. Linear transformations and activation functions are applied to these features. The contribution of each modal feature is quantified using an energy function, and the attention weights are dynamically adjusted.

In the cross-modal feature fusion stage, this paper introduces the EGAF framework, which consists of several key steps in its core processing flow. First, the feature fusion module splices and further processes the features from the two modalities. An energy weight matrix is then introduced to generate more discriminative fused features. The objective of the encoding-fusion-decoding process is to map the fused feature representation to the final classification result. A flowchart illustrating the feature fusion process is presented in Figure 5.

The specific steps are as follows:

Take the training sample $x^{s}$ as an example. Let its time-frequency image features and natural resonance frequency features be $F_{\mathrm{img}}=f_{\theta }(x^{s})\in R^{256}$ and $F_{\mathrm{seq}}\in R^{256}$, respectively. The feature fusion module combines the features from both modes to construct the multimodal features of the sample. We can express this relationship with the following formula:

(5) $$F_{\mathrm{concat}}=\left[F_{\mathrm{img}};F_{\mathrm{seq}}\right]\in R^{512}$$

where $f_{\theta }$ denotes the time-frequency analysis with a local feature dimension of 256, where the time-frequency image features $F_{\mathrm{img}}$ of the samples are extracted by the image encoder and the natural resonance frequency features $F_{\mathrm{seq}}$ are extracted by the data encoder. The aforementioned fusion features $F_{\mathrm{concat}}$ are utilized to generate more discriminative multimodal fusion features through a two-layer fully connected network. The feature vectors are mapped to the category space via a fully connected layer, resulting in the output of initial score vectors for each category.

It is important to note that the time-frequency image serves as a visualization of the energy distribution of a signal across both time and frequency dimensions. Consequently, the two parameters σ,ω of the natural resonance frequency—representing the oscillatory decay rate and the oscillation frequency of the signal, respectively—do not directly reflect changes in energy. To more comprehensively describe the physical properties of the signal and enhance the model’s ability to focus on learning the fused features, we incorporate energy information into the multimodal fusion features.

According to the theory of the singularity expansion method [20], the late-time response in the resonant region of a radar target can be expressed as the sum of the decaying negative exponents of the natural resonant frequencies. For the kth natural resonant frequency, its mode is $y_{k}(t)=r_{k}e^{(\sigma_{k}+\omega_{k}j)t}$, $r_{k}$ is the residue, $\sigma_{k}+\omega_{k}$ is the natural resonant frequency, and $\sigma_{k}$ is defined as the attenuation factor. Based on the method used to calculate signal energy $E_{k}$, its energy can be expressed as follows:

(6) $$E_{k}=\int_{0}^{\infty }{\left|y_{k}(t)\right|}^{2}dt$$

Substituting the expression of $y_{k}(t)$ into the above equation, we can get:

(7) $$E_{k}=\int_{0}^{\infty }{\left|r_{k}e^{(\sigma_{k}+\omega_{k}j)t}\right|}^{2}dt={\left|r_{k}\right|}^{2}\int_{0}^{\infty }e^{2\sigma_{k}t}dt$$

Since the stability of the system requires that $\sigma_{k}<0$, the convergence of the above integrals, the energy can be further simplified to $E_{k}=\frac{{\left|r_{k}\right|}^{2}}{-2\sigma_{k}}$. This result demonstrates that the energy of each natural resonant frequency mode is proportional to the square of its amplitude and inversely proportional to the absolute value of the decay rate. Consequently, we can utilize the energy of each mode as a weight matrix for subsequent signal analysis and feature extraction.

In the text, we can deduce that the total energy of the signal can be obtained by summing the energies of all modes:

(8) $$E=\sum_{k=1}^{N}E_{k}$$

The energy weight matrix E is introduced to characterize the energy distribution of the features, and the energy-guided modal saliency weights μ are generated through the differentiable attention mechanism, the formula is constructed in the paper:

(9) $$\mu =\mathrm{softmax}(E\cdot F_{\mathrm{concat}}^{T})$$

This weight dynamically modulates the fusion feature constructed in the paper:

(10) $$F_{\mathrm{energy}}=\mu ⊙F_{\mathrm{concat}}$$

The energy of each natural resonant frequency mode is represented as a matrix of weight coefficients. Modes with larger amplitudes and slower decay rates contribute more energy to the signal and, therefore, are assigned higher weights in the weight matrix. This approach intuitively reflects the relative importance of the various natural resonant frequency modes within the signal and facilitates the subsequent model’s ability to capture and learn the key features of the signal.

### 2.3. N-Way K-Shot Learning

In light of the rapid advancements in deep learning technology, the research paradigm for FSL has gradually transitioned towards the N-way K-shot learning problem, with multi-categorization as the target task. The N-way K-shot learning problem posits the existence of N new categories within the target task, each comprising K annotated samples (typically, K is a small value). The model is tasked with learning from these limited annotated samples and recognizing the N new categories. Specifically, an FSL task with K samples across N categories consists of a support set $D_{train}={\left\{\left(x_{i}^{S},y_{i}^{S}\right)\right\}}_{i=1}^{N_{s}}$ and a query set $D_{test}={\left\{\left(x_{i}^{q}\right)\right\}}_{i=1}^{N_{q}}$. The support set contains labeled samples used for model training, while the query set comprises unlabeled samples utilized for model testing. It is essential to note that both the support set and the query set exist within the same category space, denoted by $D_{train}\cap D_{test}=\emptyset$. $C_{novel}$ signifying the N new categories introduced in the context of the FSL task. This task setting illustrates the expanded scope of FSL, significantly enhancing the model’s ability to generalize under conditions of limited data.

The introduction of the N-class K-sample learning framework facilitates a more systematic evaluation of a model’s classification performance with a limited number of samples. This framework establishes clear objectives and evaluation criteria for the design and optimization of FSL algorithms. By utilizing a reduced number of samples, it can more accurately simulate the learning of new categories in real-world scenarios. Furthermore, incorporating multiple categories is essential for ensuring the diversity and complexity of the task, thereby preventing the model from failing to learn all categories of data during the training process.

### 2.4. Metric Space Optimization and Loss Function Design

The triplet loss function [21], initially proposed by the Google research team, establishes explicit category decision boundaries within its learned feature space by enforcing the constraint that the feature distance from an anchor to a positive sample is less than the distance from the anchor to a negative sample, within a predefined margin. In the context of small sample learning, samples from the support and query sets are utilized to construct triplets (Anchor, Positive, Negative). The Anchor denotes the feature representation of a sample, while the Positive represents a positive sample, i.e., the feature representation of another sample belonging to the same category as the Anchor. Conversely, the Negative represents a negative sample, i.e., the feature representation of a sample belonging to a different category than the Anchor. For each training batch, a set of triplets (A,P,N) is selected, and a neural network is employed to learn the embedding function and compute the feature embedding vectors. The gradient is backpropagated, and the parameters are updated to minimize the loss.

The computational method of using Euclidean distance to measure triplet loss evaluates the similarity of features through high-order operations. This approach primarily focuses on interpolating the probability distribution functions of the corresponding points while neglecting the geometric properties of the two types of feature distributions. In contrast, the Wasserstein distance metric [22] offers a more sophisticated approach by taking into account the geometric properties between probability distributions. This allows for a more accurate measurement of the distance between two feature distributions while preserving their underlying characteristics. As a result, the Wasserstein distance is chosen as the similarity metric for time-frequency image features.

When the characteristic probability distributions of ${\overset{⌢}{Q}}_{x,y},{\tilde{Q}}_{x,y}$ are, respectively, $\overset{⌢}{\kappa },\tilde{\kappa }$, the distance metric [22] is calculated as:

(11) $$W\left[{\overset{⌢}{Q}}_{x,y},{\tilde{Q}}_{x,y}\right]=\inf_{\rho \in \Pi \left(\overset{⌢}{\kappa },\tilde{\kappa }\right)}E_{{\overset{⌢}{q}}_{x,y},{\tilde{q}}_{x,y}∼\rho }\left[\left\|{\overset{⌢}{Q}}_{x,y}-{\tilde{Q}}_{x,y}\right\|\right]$$

In this context, ρ is the joint distribution of $\overset{⌢}{\kappa },\tilde{\kappa }$, Π is the set of all possible joint distributions, and ${\overset{⌢}{q}}_{x,y},{\tilde{q}}_{x,y}$ are the variables randomly selected from ${\overset{⌢}{Q}}_{x,y},{\tilde{Q}}_{x,y}$, respectively. The Wasserstein distance has the capacity to measure and optimize the difference between the two types of feature distributions. In cases where the two feature distributions exhibit a significant difference or incomplete overlap, effective gradient information can be computed, positively impacting the stability of the model optimization process. Consequently, the triplet loss function [21] is computed as follows:

(12) $$L_{Triplet}=\frac{1}{N}\sum_{i=1}^{N}\max(0,W(\varphi_{i},{\hat{\varphi }}_{i}^{+})-W(\varphi_{i},{\hat{\varphi }}_{i}^{-})+\alpha )$$

α is the interval margin, which specifies the minimum required separation for the distance between positive and negative sample pairs. max(0,⋯) indicates that the loss is non-negative and is 0 when the model satisfies the desired condition, otherwise a loss greater than 0 indicates a penalty to the model.

The DTW [23] methodology is utilized to calculate the similarity between two sequences. Given that the natural resonance frequency of the target can vary with changes in sequence length, DTW is employed as a loss function. This function has the capacity to regularize and align the two sequences, thereby reducing the impact of delays and fluctuations. Furthermore, it can be utilized to enhance the discriminative properties of the features. The sequence feature similarity measure $L_{sim\_array}$, $S_{i}$ is defined as the sequence feature of the ith sample, and P is the set of indices of samples that are similar to the sample. The minimum path distance between similar samples is then calculated as [23]:

(13) $$L_{sim\_array}=\frac{1}{N}\sum_{i=1}^{N}\sum_{j\in P}DTW(S_{i},S_{j})$$

The cross-entropy loss function [24] is a widely used metric in deep learning classification tasks, designed to assess the accuracy of the model outputs by quantifying the difference between the predicted distribution and the true distribution. According to [24], when the number of categories is N, the cross-entropy is calculated based on the true label $l_{i}$ and the model’s predicted probabilities ${\hat{l}}_{i}$:

(14) $$L_{CE}=CrossEntropyLoss=-\sum_{i=1}^{N}l_{i}\cdot \log({\hat{l}}_{i})$$

**Figure 6:**
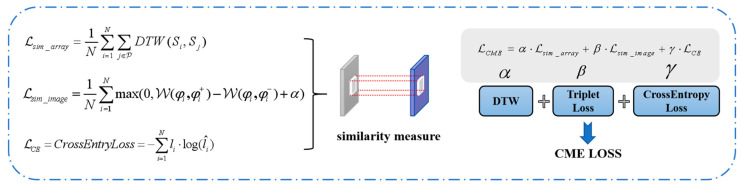
Metric space optimization and loss function design are integrated with contrastive learning and classification supervision, allowing for simultaneous optimization of modal alignment and feature discrimination.

The sequence feature similarity metric, image feature similarity metric, and categorical cross-entropy loss are integrated into the CME loss. The similarity metric can be viewed as an implicit regularization term, designed to prevent the model from overfitting to unimodal features. By constraining the similarity within the multimodal feature space, the similarity metric aids the model in learning a shared representation across modalities, thereby enhancing the complementarity of intermodal information. Simultaneously, the cross-entropy loss serves as a mechanism to ensure the model’s discriminative performance in classification tasks, thus preventing any deviation from the intended objective. The equation is derived in the paper:

(15) $$L_{CME}=\alpha \cdot L_{sim\_array}+\beta \cdot L_{sim\_image}+\gamma \cdot L_{CE}$$

where α, β and γ are the weight coefficients, currently set to 1:2:7, in order to achieve a synergistic optimization of classification loss and feature regularization. Initially, the focus is on modeling feature distribution, where the divisibility of the time-frequency image features is superior to that of the natural resonance frequency features. The coefficients α, β are set to 0.1 and 0.2, respectively. The subsequent enhancement of the classification boundary optimization is based on the classification task being the primary objective, with a coefficient γ set to 0.7. Here, the term $L_{sim\_array}$ represents the sequence feature similarity loss, while $L_{sim\_image}$ denotes the image feature similarity loss. The loss function $L_{CME}$ is defined as the weighted sum of the classification loss and the triplet loss, and this composite approach optimizes both classification performance and feature representation. The schematic illustrating the metric space optimization and loss function design is presented in Figure 6.

## 3. Methods and Data

### 3.1. Experimental Dataset Construction

**Figure 7:**
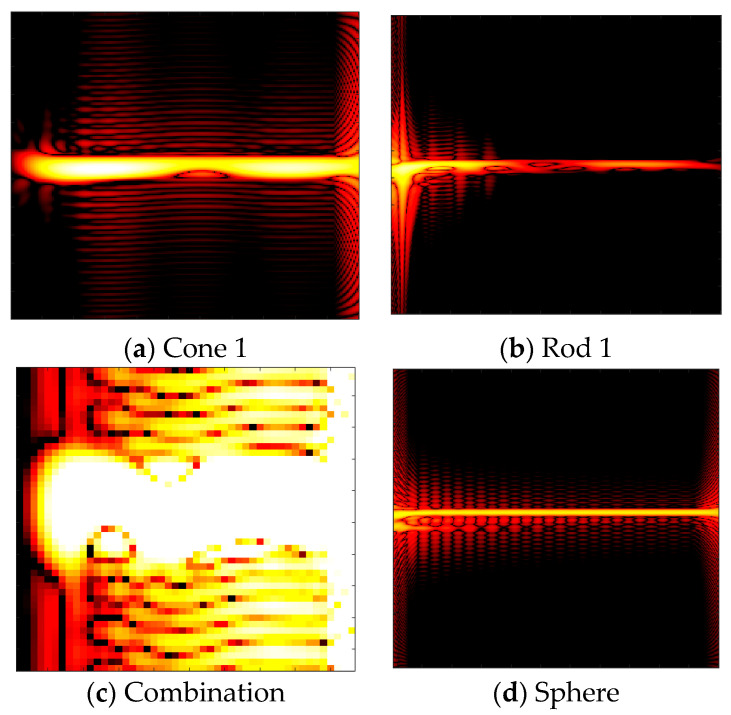
CWD time-frequency images of cone 1, rod 1, combination, and sphere reveal distinct spectral characteristics. The spectra of cone 1 and rod 1 are relatively concentrated, indicating that the frequency components of their echo signals are singular and stable. In contrast, the sphere exhibits a slightly wider spectrum, likely due to the geometric symmetry of the target. The spectrum of the combination is the most complex, reflecting the interactions among multiple targets or target components.

In order to verify the effectiveness of the method proposed in this paper, and due to the limited availability of publicly accessible signal echo datasets within the resonance band range, we constructed the necessary small sample dataset using the FEKO 2021 Version simulation method [25]. First, we simulated a batch of simple models, which included nine types of targets, such as spheres, cones, rods, and cubic panels, all made of copper. The parameters of the simulated models are listed in Table 1 below:

In far-field, single-station conditions, the frequency range is set from 1 MHz to 2.7 GHz, with the frequency points configured for sweeping. The observation angle interval is set to 5°, and the angle variation range is $\theta \in \left[0,180{}^{\circ}\right],\varphi \in \left[0,90{}^{\circ}\right]$. The frequency domain signal of the target is obtained using the Method of Moments, and the corresponding time domain echo is derived through the inverse fast Fourier transform (IFFT). Time-frequency analysis is performed using the Choi–Williams distribution (CWD) based on this echo. The CWD transformation formula [26] is as follows:

(16) $$CWD_{x}(t,\omega )=\iint \sqrt{\frac{\sigma }{4\pi \tau^{2}}}A_{x}(\mu ,\tau )e^{\sigma {\left(u-t\right)}^{2}/4\tau^{2}}\cdot e^{-j\omega \tau }d\mu d\tau$$

where t is the time, ω is the angular frequency; σ is the attenuation coefficient, which determines the filter bandwidth and is set to 1 in this paper to balance the cross-talk with the signal resolution. τ is the time delay, and is the variable of integration. $A_{x}(\mu ,\tau )=x(\mu +\frac{\tau }{2})x^{*}(\mu -\frac{\tau }{2})$ is the instantaneous autocorrelation of the signal. The CWD time-frequency images of the four targets cone 1, rod 1, combination and sphere are given in Figure 7 below.

**Figure 8:**
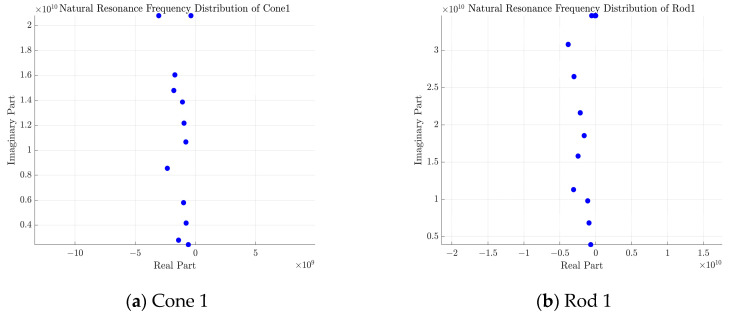

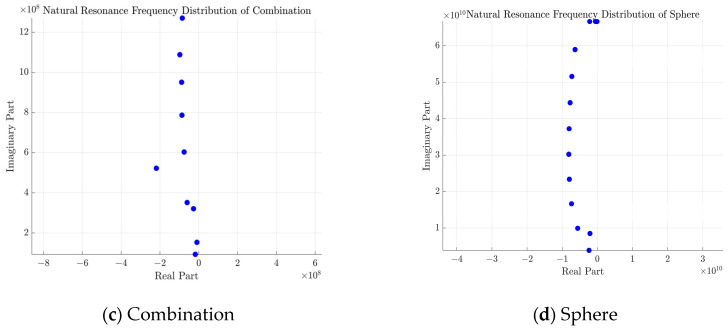
The natural resonance frequency distribution of cone 1, rod 1, the combination, and the sphere reveals distinct characteristics. The frequency distribution of cone 1 and rod 1 is linear, indicating that their resonant modes are regular. In contrast, the distributions of the sphere and the combination are more complex, reflecting their geometric and structural intricacies.

The natural resonant frequencies were extracted using the joint matrix bundle method as described in [27]. A Hankel matrix $H_{k}$ is constructed from the time-domain signals y(t) obtained at each angle, and the natural resonance frequencies of the joint matrix are determined by solving for the generalized eigenvalues of the singular value decomposition. Figure 8 below illustrates the distribution of natural resonance frequencies for cone 1, rod 1, the combination, and the sphere. The horizontal axis represents the real part, while the vertical axis represents the imaginary part.

A total of nine types of targets and 2019 samples were generated in the dataset. The training and test sets were divided in an 8:2 ratio, with the model randomly selecting 1615 samples for the training set and designating the remaining 404 samples for the test set. This division facilitates the evaluation of the model’s performance. A 5-way 1-shot few-shot categorical sampling strategy, which is commonly used, was employed to randomly select five categories from the training set, with six samples chosen from each category. As a result, each category contains five support set samples and one query set sample. The experiments were conducted in an end-to-end manner using the Stochastic Gradient Descent (SGD) optimization algorithm, with a batch size of 32, a learning rate of 0.001, a momentum of 0.9, and 100 iterations, maintaining consistent conditions throughout.

### 3.2. Evaluation Indicators

In order to measure feature separability and classification effect, we selected some common index calculation methods in deep learning and machine learning according to [28].

#### 3.2.1. Recognition Accuracy

The model’s performance in classifying all the targets is indicated by the recognition accuracy *Acc*. The model is considered to have achieved classification accuracy when the sample predictions align with the true values. The possible results of this process are as follows: TP (true positive), where the sample predictions match the true values and are all positive; FP (false positive), where the sample predictions are positive and the true values are negative; FN (false negative), where the sample predictions are negative and the true values are positive; and TN (true negative), where the sample predictions match the true values and are both negative. The average recognition accuracy is a metric of the model’s overall recognition accuracy, which is selected to assess the model’s performance. It is calculated based on the following formula:

(17) $$Acc=\frac{TP+TN}{TP+FP+FN+TN}$$

#### 3.2.2. Stability Score

The output of the model Z, represents the score of each sample belonging to each class, which the softmax function converts to a probability distribution:

(18) $$p_{i,j}=\mathrm{softmax}(Z)=\frac{e^{z_{i,j}}}{\sum_{k=1}^{C}e^{z_{i,k}}}$$

$z_{i,j}$ is the logit value of the ith sample in the jth class as output by the model, and $p_{i,j}$ is the predicted probability. C is the total number of categories. For each unique class c∈unique(labels) find the set of indexes $S_{c}=\{i\left|y_{i}=c\right.\}$ of all the samples belonging to that class, for extracting the predictive probability matrix $P_{c}={\left[p_{i,j}\right]}_{i\in S_{c}}$ of its samples as shown in Equation (19):

(19) $$Var_{c,j}=S\left[{\left(P_{c,j}-\mu_{c,j}\right)}^{2}\right]$$

where $\mu_{c,j}=S[P_{c,j}]$ is the probability mean of the samples of class c over the predicted class j. For each true class c, calculate the mean of all predicted class variances:

(20) $$Stability_{c}=\frac{1}{C}\sum_{j=1}^{C}Var_{c,j}$$

The mean of all true class stability scores was calculated to obtain the final results:

(21) $$Stability=\frac{1}{\left|Classes\right|}\sum_{c\in Classes}Stability_{c}$$

It can be seen that when the class stability score is lower, it proves that the class features are more stable.

#### 3.2.3. Intra-Class Average Distance and Inter-Class Separation Metrics

Intra-class average distance was used to measure the degree of clustering of samples within the same class in the feature space and is calculated as follows:

(22) $$Intra-class=\frac{1}{C}\sum_{c=1}^{C}\frac{1}{N_{c}(N_{c}-1)}\sum_{i,j\in c,i\neq j}{\left\|f_{i}-f_{j}\right\|}_{2}$$

where $N_{c}$ is the number of samples of the cth class and $f_{i}$ is the eigenvector of the ith sample.

Inter-class separation metrics was measured by the ratio of the mean distance between the sample centers of different classes to the inter-class variance.

(23) $$Inter-class=\frac{\sum_{c1\neq c2}{\left\|\mu_{c_{1}}-\mu_{c_{2}}\right\|}_{2}}{C(C-1)\sqrt{\frac{1}{C}\sum_{c=1}^{C}\sigma_{c}^{2}}}$$

where $\mu_{c}$ is the mean of the cth class feature and $\sigma_{c}^{2}$ is the intra-class variance.

The ratio of inter-class separation metrics to intra-class average distance was used as an indicator of the separability score, and the formula was calculated as follows:

(24) $$Divisibility-score_{F_{1}}=\frac{Inter-class_{F_{1}}}{Intra-class_{F_{1}}}$$

Thus, the rate of change of separability score from feature $F_{1}$ to feature $F_{2}$ is:

(25) $$\frac{Divisibility\_score_{F_{1}}-Divisibility\_score_{F_{2}}}{Divisibility\_score_{F_{1}}}\times 100\%$$

## 4. Results and Discussion

### 4.1. Effect of Different Features on Recognition Results

In order to investigate the effects of various features on recognition results, unimodal time-frequency, unimodal sequence, and concatenated fusion features are utilized as inputs for the comparative recognition experiments. By maintaining constant parameters across 100 experiments, the mean outcomes following 100 iterations are calculated.

As demonstrated in Table 2, the fusion features employed in this study exhibit optimal performance in terms of recognition accuracy and stability. In scenarios requiring high accuracy, these features remain the preferred choice, despite the increased training and inference time associated with their more complex model structure. When unimodal time-frequency features are utilized as input, the performance is more balanced regarding accuracy and inference time, with a reduction in inference time of 17.3%. The fusion feature achieves a recognition accuracy of 95.36%, while the unimodal time-frequency feature attains 93.10% recognition accuracy, with the former exceeding the latter by 2.26%.

Despite the advantages of the unimodal sequence feature model, such as its compact data size and rapid computation, its performance in few-shot learning (FSL) remains suboptimal. This is primarily due to the lack of a significant clustering effect in the feature distribution of natural resonance frequency data, combined with minimal inter-class variability, which impedes the model’s ability to learn the underlying physical properties. As a result, the unimodal sequence feature achieves only 64.18% recognition accuracy. In contrast, the performance of the concatenation fusion feature model is consistent across all metrics. The direct tensor concatenation of time-frequency and sequence data is straightforward and intuitive; however, time-frequency data and structured sequence data exist in different feature spaces and convey distinct semantic information. Consequently, it is challenging for the model to capture the correlation between the two modalities. The direct concatenation of features lacks clear physical meaning, resulting in a lower recognition accuracy rate. In comparison to the 86.63% recognition rate achieved with concatenation features, the recognition rate of the fusion features we employed increased by 8.73%. A comparison of the parametric metrics indicates that the features utilized in this study demonstrate only a marginal improvement over the use of unimodal time-frequency features combined with concatenation fusion features, while the FLOPs remain constant. This observation supports the conclusion that the model’s resource consumption and computational complexity experience negligible increases.

**Figure 9:**
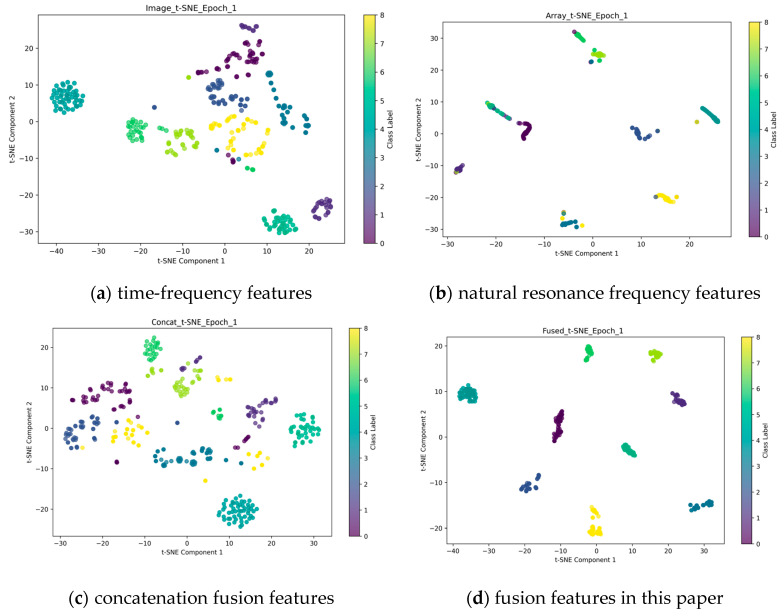
Distribution of t-SNE feature visualizations.

As demonstrated in Table 3, the results of the quantitative analysis reveal significant disparities in the spatial characteristics of the various features, as indicated by the intra-class average distance and inter-class separation metrics. The intra-class average distance for unimodal time-frequency features is 5.72, while the inter-class separation metric reaches 21.81. This suggests a moderate degree of intra-class aggregation but limited inter-class differentiation capability. In contrast, the unimodal sequence features exhibit excessive compression, with an intra-class average distance of only 0.10 and an inter-class separation metric as low as 0.88. This indicates a deficiency in effective separability within the original space. The simple concatenation fusion feature maintains an intra-class distance of 5.04, but the inter-class separation metrics plummet to 13.36, revealing that the direct concatenation of different modal features may trigger semantic conflicts and does not have good consistency. In contrast, the multimodal fusion method proposed in this paper enhances the inter-class separation to 25.83 while maintaining the intra-class distance metrics steady. This leads to an improvement in separability scores of 18.37% and 70.19% compared to unimodal time-frequency features and concatenation fusion features, respectively. This phenomenon can be attributed to the energy-guided cross-modal fusion mechanism, which establishes the optimal mapping between the support set and the query set through Wasserstein distance constraint. Furthermore, the fused features of this paper’s method enhance the boundary identifiability while maintaining the intra-class compactness. The visualization distribution of different features after t-SNE, as presented in Figure 9, also substantiates the aforementioned conclusion. In comparison with the other three features, the fused features in this paper exhibit enhanced divisibility and aggregation.

**Figure 10:**
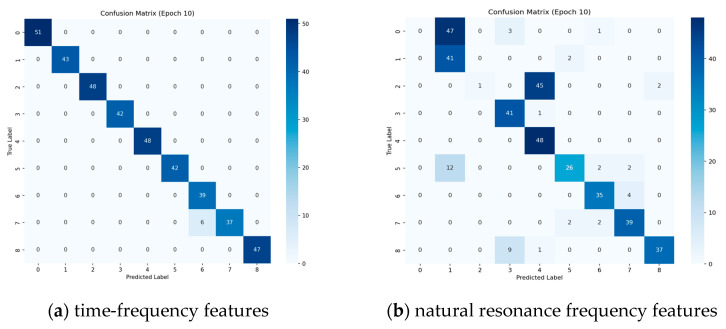

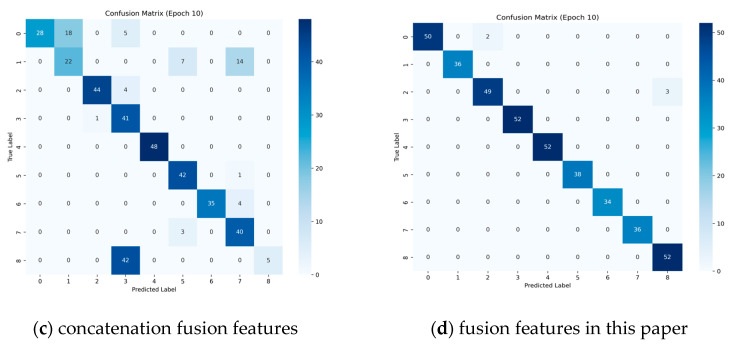
Confusion matrix of experimental results for different features.

As demonstrated in Figure 10, an investigation into the differential performance of different features in different target recognition tasks is presented in the confusion matrix analysis (Epoch10). The unimodal time-frequency features exhibit interclass confusion for geometrically similar targets, such as rod 1 and rod 2, with a recognition accuracy of 86.05% for rod 2, attributable to the high similarity of the distribution of scattering centers in the time-frequency image. The fusion feature proposed in this paper demonstrates superior inter-class discrimination; however, it still exhibits a misclassification rate of 5.77% for targets exhibiting similar lateral scattering characteristics, such as combination and cubic panels. The physical mechanism underlying this phenomenon can be attributed to the presence of certain overlapping distributions in the natural resonant frequency attenuation factor for these targets. The utilization of sequence and concatenation features has been demonstrated to be ineffective, as both of these approaches appear to lack the capacity to differentiate between rod targets of varying dimensions. Further analysis indicates that a single natural resonant frequency sequence feature is inadequate in corresponding to variations in target size, and it is challenging to differentiate between similarly shaped rod targets. Additionally, projected similarity at characteristic observation angles triggers confusion in the frequency-domain features. For instance, similarly shaped targets projected perpendicularly to the radar line-of-sight direction, such as cones and spheres, are also not accurately recognized. The direct concatenation of features disregards the semantic gap between modalities, and the confusion matrix presents diffuse misclassification and generalized confusion for multi-class targets. This indicates that simple feature superposition does not possess satisfactory feature discriminative properties.

### 4.2. Effect of Different Sequence Lengths on Recognition Results

In order to investigate the effect of different natural resonance frequency sequence lengths on the model performance, based on the energy weighting coefficient matrix assignment strategy in Section 2.2, the natural resonance frequencies with larger weighting coefficients are selected as the input sequence data. The approach we take is not to deal with the sequence length, directly input the network. It is acknowledged that natural resonance sequences of varying lengths are associated with different targets; therefore, the natural resonance frequencies with lengths of 3, 5, and 11 are selected in accordance with the ranking relationship of decreasing weight coefficients to form new sequence data. The identification results are then compared in the experiments. Maintaining constant all other parameters in 100 experiments, the mean of the results following 100 iterations is calculated.

The comparative experimental results in Table 4 reveal the intrinsic correlation between the energy-dominated natural resonance feature selection mechanism and data missing robustness. In addressing the issue of excluding spurious data during the extraction of natural resonance frequencies, this study incorporates the disparity in energy distribution between authentic resonance modes and spurious interference. Authentic natural resonance frequencies are characterized by higher energy and more stable distribution, while spurious data exhibits low energy. It is important to note that without altering the sequence length, this corresponds to complete resonant mode coverage. However, the presence of spurious data can potentially influence the outcomes. When the sequence length is 11, the natural resonance frequency corresponding to certain angles is already absent, yet the recognition accuracy reaches 96.90%, indicating that the high-dimensional sequence can adequately capture the intrinsic resonance characteristics of the target. Furthermore, the feature selection method based on the decreasing energy order effectively strengthens the contribution of the dominant modes, which in turn improves the regularity of the feature distribution. However, when the sequence length is reduced to 5 and 3, the recognition effect is diminished by the influence of missing data, and the recognition accuracy drops to 92.38% and 93.26%, respectively. This may be due to the fact that the sequence is too short with missing modes, which cannot completely characterize the multi-resonance effect, resulting in the increase in intra-class feature variance. However, the model maintains more than 90% recognition accuracy even under severe data-missing conditions, thereby verifying the effectiveness of the multimodal complementary mechanism. The time-frequency image features can be discriminatively compensated by time-domain energy focusing, while the BiLSTM sequence encoder effectively suppresses spurious modal interference. The experiment demonstrates the method’s feasibility in scenarios where natural resonant frequencies are incomplete, offering a valuable reference point for target recognition in practical applications.

### 4.3. Validation of the Effectiveness of the Loss Function

In order to explore the effect of loss function on model performance, different loss functions are used for comparison experiments. Cross-entropy + triplet loss, cross-entropy loss, triplet loss, and contrast loss are utilized as loss functions, respectively. One hundred repetitions of experiments under the same conditions are conducted to calculate the average recognition accuracy and stability scores.

As demonstrated in Table 5, a comparison of disparate loss function models reveals that the CME loss function in this paper attains the maximum recognition accuracy. Cross-entropy loss is a frequently employed loss function in classification tasks, which is capable of effectively distinguishing between different classes and exhibits stable classification performance. However, it solely optimizes inter-class decision boundaries during training and is deficient in its capacity to characterize multimodal feature distributions. Despite the fact that the triplet loss is both small in variance and fast, its optimization objective is not fully consistent with the classification task. As a separate loss function, it lacks explicit classification supervision, which causes the model to fall into a local optimum. The low variance reflects the risk of possible over-compression of the feature space. When the cross-entropy loss is combined with the triplet group loss to construct the hybrid loss, although the stability is improved by the feature space constraint, the accuracy is not significantly improved due to the conflicting gradient directions of the two different losses. The variance of the contrast loss is 0, and the recognition effect is very poor, probably because its optimization objective does not match the classification task and the model fails to learn effectively. The experimental results demonstrate that CME lOSS exhibits enhanced robustness and attains synergistic optimization of classification loss and feature regularization through the weight adjustment mechanism. With a weight factor set to 3:7, this strategy prioritizes feature distribution modeling during the initial stage and strengthens the optimization of classification boundaries during the subsequent stage. This phased optimization approach maintains high efficiency while adapting to the multimodal feature distribution.

### 4.4. Validation of the Effectiveness of EGAF Framework

In order to investigate the impact of the energy bootstrapping strategy described in Section 2.2 of this paper on the model effectiveness, comparison experiments are conducted using unprocessed fusion features and competence-boosted fusion features, respectively. A total of 100 repetitions of the experiments are conducted under identical conditions, and the mean recognition accuracy and stability scores are calculated.

As demonstrated in Table 6, the EGAF framework employed in this paper offers distinct advantages in enhancing recognition accuracy and stability. The recognition accuracy is notably higher when the EGAF framework is implemented compared to its absence, though its performance is less stable. The incorporation of the energy matrix within the model facilitates the concentration on the energy aggregation region within the time-frequency domain, which corresponds to the primary modes of the target resonance. This, in turn, ensures that the deep fusion features are spatially consistent with the physical scattering mechanism. The region of energy concentration can be mapped to the critical scattering center of the target, which is consistent with the physical perception convention of the model. The time-frequency image is indicative of the spatial energy distribution, and the natural resonance frequency is reflective of the transient oscillation characteristics. It can be posited that the two features reflect the steady state and transient characteristics of the target scattering, respectively, to a certain extent. The energy-guided strategy has been shown to constrain the feature search space, thereby enabling the model to converge more rapidly to achieve optimal classification results. This is advantageous in scenarios involving small sample sizes; however, it should be noted that this approach may also result in a concomitant increase in instability during the convergence process.

### 4.5. Validity Verification of Training Strategies

To investigate the impact of loss functions on model performance, comparative experiments were conducted using different training strategies. The optimizer selected for this study is SGD with Momentum, while Adam and RMSProp optimization methods were employed to perform 100 repeated experiments under identical conditions. The average recognition accuracy and stability scores were then calculated.

It can be seen from Table 7 that the optimizer combination of SGD + Momentum adopted in this paper performs best in recognition accuracy, possibly because the introduction of Momentum helps the model to build an inertial system with gradient updates and converge to a better local optimal solution faster. The Adam optimizer uses a fixed learning rate, so the accumulation of deviation at the first-order distance will lead to the late update step size being too small, which is the best performance in training stability, but the fast convergence will cost the model capacity, and the recognition accuracy is slightly worse than SGD + Momentum. RMSProp uses the exponential moving average of the squared gradient to adjust the learning rate, which is not stable enough. It may be that the long-term gradient memory and the instantaneous gradient in multi-modal features fluctuate and conflict, and the gradient magnitude difference between feature channels will be amplified, so the recognition accuracy is lower than that of other optimizers.

## 5. Conclusions

In order to solve the problems of insufficient separability of single features and limited generalization ability of models in radar target recognition tasks under small sample conditions, Res-BiLSTM-MFCnet fused with time-frequency-resonance features is proposed. By deeply fusing the features of time-frequency images and natural resonant frequency sequences and combining the similarity measures of two features with the CME loss from cross-entropy loss, the model learns a feature representation space with significant class discrimination. The recognition accuracy of 95.36% is achieved on the simulation dataset, which is significantly improved compared to the traditional single-modal method. The core values can be summarized as follows:

1. The natural resonant frequencies, which characterize the scattering properties of the resonant region, are integrated into the FSL framework. By combining features from both the time-frequency domain and frequency domain, the model effectively captures the intrinsic physical characteristics of the target while mitigating overfitting issues associated with low sample complexity.
2. A Res-BiLSTM-MFCNet architecture, built upon ResNet-18 and BiLSTM, is developed to achieve a deep integration of time-frequency images and sequential features. Experimental results demonstrate that the separability score of the fused features in the metric space has increased by 18.37%, thereby validating the complementary advantages inherent in multi-modal approaches. Additionally, the EGAF framework is introduced to transform the amplitude and decay rate of natural resonant frequencies into a feature weight matrix that represents energy, further enhancing the representational capacity of these features.
3. In light of the multimodal fusion mechanism and classification requirements, a composite optimization objective function, namely CME loss, is devised. This function integrates the optimization objectives of multimodal feature similarity measurement and cross-entropy loss. By doing so, it not only enhances the discriminability of features but also ensures classification performance. As a result, it furnishes a novel optimization paradigm for multimodal FSL.

Nevertheless, several limitations persist in this study. The present experiments rely solely on simulated data and fail to account for factors such as multipath interference within the measured environment. Future research endeavors should be directed towards enhancing the model’s anti-interference capabilities.

## Figures and Tables

**Table 1:** Simulation model parameters.

Graph	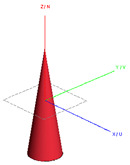	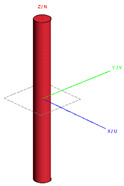	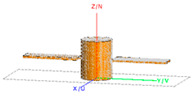	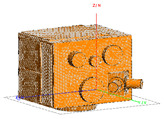	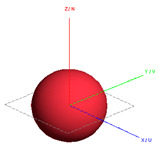	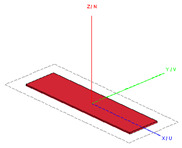
Name	cone 1/2	rod 1/2/3	combination	cuboid	sphere	cubic panel
Size	radius 0.075/0.272 heights 0.5/1.81	radius 0.005/0.015/0.03 heights 1.0/0.3/0.6	lengths 8 (includes side panels)	lengths 0.685 widths 0.515 heights 0.524	radius 0.078 m	lengths 1 widths 0.25 heights 0.01
Data volume	259/181	181/259/259	189	173	259	259

**Table 2:** Results of comparison experiments with different features.

Features	Acc/%	Stability	Inference Time/s	Quantity of Participants/M	FLOPs/G
unimodal time-frequency feature	93.10 ± 0.36	0.0049	17.95	11.34	2.28
unimodal sequence	64.18 ± 0.51	0.0111	2.82	0.20	0.01
concatenation fusion	86.63 ± 0.25	0.0069	22.93	11.51	2.28
fusion in this paper(ours)	**95.36 ± 0.22**	**0.0005**	**30.17**	**11.67**	**2.28**

The bolded results represent our method and are the same in the other tables.

**Table 3:** Intra-class average distance and inter-class separation metrics for different features.

Features	Intra-Class Average Distance	Inter-Class Separation Metrics	Separability Score
Unimodal time-frequency	5.72	21.81	3.81
Unimodal natural resonance frequency	0.10	0.88	4.4
Concatenation fusion	5.04	13.36	2.65
Fusion in this paper (ours)	**5.73**	**25.83**	**4.51**

**Table 4:** Comparison results of different sequence lengths.

Sequence Length	Acc/%	Stability
3	93.26 ± 0.35	0.0006
5	92.38 ± 0.27	0.0004
11	96.90 ± 0.21	0.0003
Methods used in this paper	**95.36 ± 0.22**	**0.0005**

**Table 5:** Comparative experimental results of different loss function models.

Loss Function	Acc/%	Stability
Cross-entropy + triplet loss	91.03 ± 0.45	0.0098
Cross-entropy loss	91.03 ± 0.51	0.0089
Triplet loss	70.34 ± 0.11	0.0001
Contrast loss [29]	27.59 ± 0.01	0
CME loss (ours)	**95.36 ± 0.22**	**0.0005**

**Table 6:** Results of experiments comparing models with and without EGAF framework.

Whether or Not to Use the EGAF Framework	Acc/%	Stability
No EGAF framework used	93.80 ± 0.09	0.0003
Use EGAF framework	**95.36 ± 0.22**	**0.0005**

**Table 7:** Comparative experimental results of different optimizers.

Optimizer Type	Acc/%	Stability
Adam optimizer	91.03 ± 0.16	0.0002
RMSProp [30]	88.28 ± 0.27	0.0016
SGD + Momentum (ours)	**95.36 ± 0.22**	**0.0005**

## Data Availability

Data will be made available on request.
